# Decorations for Hospital Secretaries

**Published:** 1916-11-25

**Authors:** 


### Decorations for Hospital Secretaries.

*To the Editor of* The Hospital.

Sir,—Your leading article on the subject of Knighthoods for Hospital Secretaries, and the comments on it which appeared in your succeeding issues (The Hospital, November 4, p. 78, and November 11, p. 127), have raised a topic which touches the members of the higher branches of hospital administration very closely.

In London alone the voluntary hospitals can supply two instances, at least, of hospital officials decorated in connection with services rendered to their institutions and so to the public—namely, Sir Cooper Perry, of Guy's, and Mr. Michelli, C.M.G., of the *Dreadnought* Hospital. So that there is not quite such a dearth of precedent in the matter as many hospital administrators seem to think. Still, one may grant readily enough that such State recognition of hospital officers is rare; and it is also easy to agree with your correspondent "Hospital Secretary" that in view of the frequent opportunities of wire-pulling which the secretaries and superintendents of large hospitals enjoy, the fact that these are not made use of redounds to their credit. In this country the whole allocation of orders and decorations is hopelessly illogical and corrupt. From a peerage down to the non-official decorations of the Order of St. John of Jerusalem, any mark of what is supposed to be the State's appreciation of good work done for the common weal can be bought for money. The tariff for the various steps of the peerage, for baronetcies, for knighthoods, and for orders of all kinds is well known; and these "distinctions" can be purchased by anyone who will pay the regular price for them to the campaign funds of the party in power. That this is the case is notorious, and has long been so, to the deep disgrace of politicians of both the principal parties. In these circumstances, which cannot be denied, it can equally well be argued that since an order is no real mark of distinction or of loyalty, or of service to the State, no hospital official need hanker after one, for he will merely find himself, when he does get one, in the company of shady plutocrats and minor political hacks; or that since any of these latter gentry can get their decorations without difficulty, there can be no possible logical reason for refusing similar "honours" to hospital secretaries. The real reform which hospital people, as citizens of an Empire with pretensions to greatness, should first strive after is the abolition of the revolting system of purchase, now openly practised in this country. When that has been brought about, it will be time to start considering what classes of workers should be entitled to have their most successful and public-spirited members recognised by the bestowal of decorations.—I am, Sir, yours faithfully,

Imperialist.

* The Hospital, November 11, p. 112.

